# PyMM: An Open-Source Python Program for QM/MM Simulations Based on the Perturbed Matrix Method

**DOI:** 10.1021/acs.jctc.2c00767

**Published:** 2022-11-15

**Authors:** Cheng Giuseppe Chen, Alessandro Nicola Nardi, Andrea Amadei, Marco D’Abramo

**Affiliations:** † Department of Chemistry, Sapienza University of Rome, Rome00185, Italy; ‡ Department of Technological and Chemical Sciences, University of Rome Tor Vergata, Rome00133, Italy

## Abstract

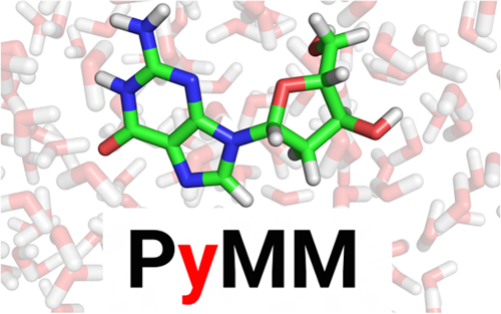

Quantum mechanical/molecular mechanics (QM/MM) methods are important tools in molecular modeling as they are able to couple an extended phase space sampling with an accurate description of the electronic properties of the system. Here, we describe a Python software package, called PyMM, which has been developed to apply a QM/MM approach, the perturbed matrix method, in a simple and efficient way. PyMM requires a classical atomic trajectory of the whole system and a set of unperturbed electronic properties of the ground and electronic excited states. The software output includes a set of the most common perturbed properties, such as the electronic excitation energies and the transitions dipole moments, as well as the eigenvectors describing the perturbed electronic states, which can be then used to estimate whatever electronic property. The software is composed of a simple and complete command-line interface, a set of internal input validation, and three main analyses focusing on (i) the perturbed eigenvector behavior, (ii) the calculation of the electronic absorption spectrum, and (iii) the estimation of the free energy differences along a reaction coordinate.

## Introduction

QM/MM methods established themselves as one of the primary tools to model biological systems, combining accuracy and computational efficiency.^1,2^ The perturbed matrix method (PMM)^3−5^ is a relatively recent QM/MM method that was developed with the aim of reducing the computing time necessary for this kind of calculation, making it suitable for application to complex systems, ranging from simple molecules in solution to protein–DNA complexes. PMM has been successfully applied to reproduce different properties such as the UV–vis spectra,^4,6,7^ fluorescence spectra,^8^ circular dichroism spectra,^6^ IR spectra,^4,9^ and redox potentials.^10,11^ The method adopts an *a posteriori* strategy, i.e., the effect of the perturbation on the QM region of interest, typically evaluated from a classical molecular dynamics, is evaluated for every configuration at a very limited computational cost. Such an approach usually requires few quantum mechanical calculations (for a rigid QM region requiring a single calculation), effectively making the time scale of the PMM simulations on par with classical MD simulations for large systems and long trajectories. Although such a remarkable gain in efficiency makes PMM an appealing method to study complex solutes in realistic environments, the lack of programs able to perform this kind of calculation limited its use. That is, PMM is currently applied using in-house software packages not availaable to the community.^12^ To address this issue, we present here the PyMM software package, a program written in Python3, that aims at making the application of the MD-PMM approach easily accessible, thus providing, at a reduced computational cost, an accurate description of the perturbed electronic properties of interest.

The MD-PMM scheme can be described as follows:

1. Perform the classical MD simulation of the system to sample an adequate region of the system conformational space.
2. Perform the quantum mechanical calculations on the Quantum Center (QC, the region of the system which we are interested in describing the electronic properties) to obtain the ground-state and excited-state electronic properties in gas phase (i.e., the unperturbed electronic properties).
3. Apply the PMM calculation for each frame sampled by the MD simulation.

**Figure 1:**
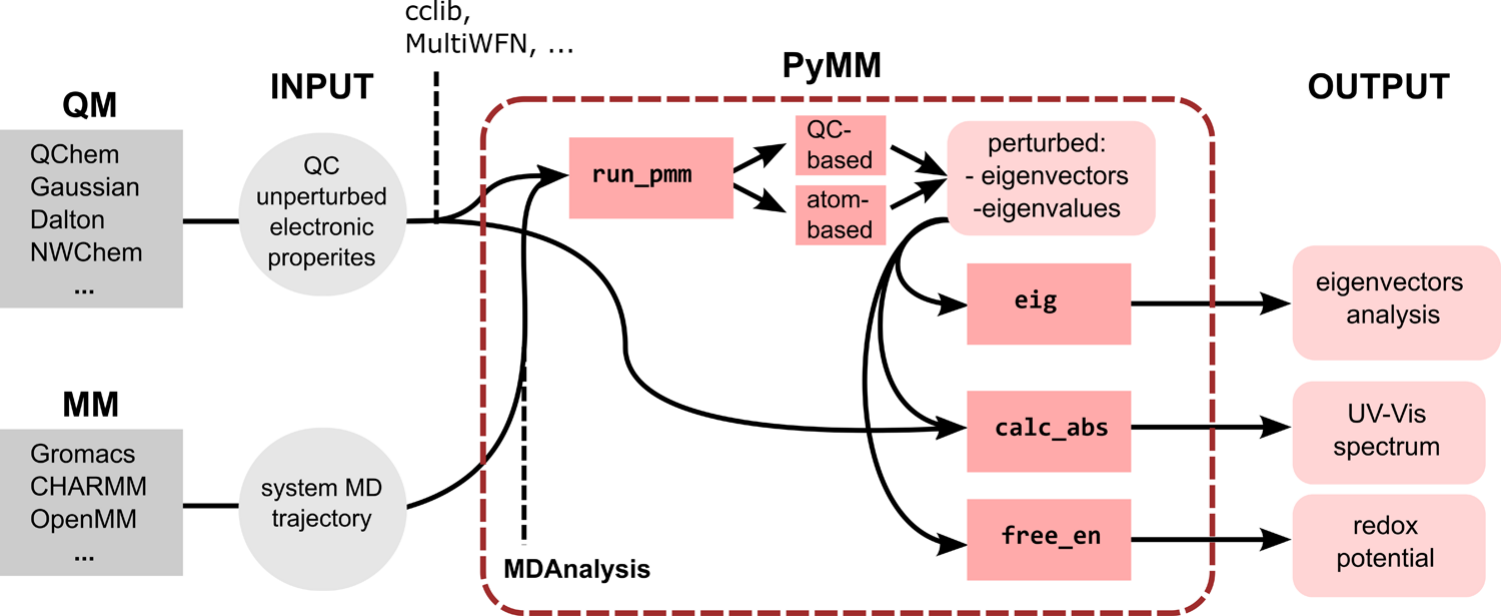
Structure of PyMM within the overall MD-PMM workflow.

PyMM focuses on the third step, while the quantum chemical calculation and the MD simulation need to be performed by the user using available software packages. PyMM relies on the MDAnalysis library^13,14^ to read the MD trajectory files; thus, all of the file formats supported by MDAnalysis are supported by PyMM as well. Numba JIT compiler^15^ was used to speed up key bottleneck steps in the calculation. Moreover, PyMM includes modules for the calculation of the UV–vis absorption spectrum, the free energy differences between electronic states and the analysis of the perturbed electronic states, with a particular focus on their dynamical behavior. All of the PyMM features can be accessed through a simple and user-friendly command-line interface. Step 1 of the MD-PMM scheme requires the user to provide MD trajectories obtained by popular software such as Gromacs,^16^ CHARMM,^17^ OpenMM,^18^ Amber,^19^ and DL_Poly.^20^ Similarly, the electronic properties of the isolated QC (Step 2) can be obtained at the desired level of theory (e.g., TD-DFT, EOM-CCSD) by means of available QM software packages such as Gaussian,^21^ Dalton,^22^ Q-Chem,^23^ and many others.^24−26^ These unperturbed electronic properties need to be extracted from the output of such programs and provided to PyMM as text files. This process can be facilitated by the use of external tools.^27,28^ The overall structure of the PyMM program and the current workflow to perform MD-PMM calculations are shown in Figure 1.

## Software Features

### MD-PMM Simulations: run_pmm Module

In the PMM approach, the electronic Hamiltonian operator *Ĥ* of the QC perturbed by the environment can be expressed as
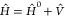
1where *Ĥ*^0^ is the electronic Hamiltonian operator of the isolated (unperturbed) QC and *V̂* is the perturbation operator describing the interaction between the QC and the environment. We can therefore obtain the Hamiltonian matrix *H̃*, with elements [*H̃*]_*l*,*l*′_, expressed on the basis set provided by the unperturbed electronic eigenstates Φ_*l*_^0^ (i.e., calculated considering the isolated QC) with eigenvalues 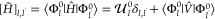
2

When PMM is coupled with classical MD simulations (MD-PMM), ⟨Φ_*l*_^0^|*V̂*|Φ_*l*′_^0^⟩ refers to the contribution arising from the interaction between the QC and the environment as provided by the MD trajectory. For each frame, such interaction is calculated to obtain *H̃*, which is then diagonalized to provide the perturbed eigenvectors Φ_*k*_, describing the QC embedded in the environment expressed in the unperturbed basis set Φ_*l*_^0^ and the corresponding perturbed eigenvalues . ⟨Φ_*l*_^0^|*V̂*|Φ_*l*′_^0^⟩ can be estimated at different levels of approximation. In particular, the perturbation exerted on the QC by its atomic-molecular environment can be treated as equivalent to a perturbing external field (i.e., the electric field provided by the atomic charge distribution as described by the charge density of the semiclassical environment). Therefore, the perturbation operator can be expressed as
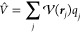
3where *j* runs over all of the relevant QC particles (i.e., electrons and nuclei), *q*_*j*_ is the charge of the j*th* particle, ***r***_*j*_ are the corresponding coordinates, and  is the electric potential produced by the perturbing environment.

PyMM currently supports MD-PMM simulations to be carried out by performing a QC-based expansion of the *V̂* operator (i.e., dipolar approximation) or by performing an atom-based expansion (i.e., by expressing the perturbation operator in terms of the perturbing field at each atom; the rigorous derivation of both the approaches can be found in a previous work^4^).

In the QC-based approach, the QC-environment, the perturbation is expanded within the dipolar approximation, and the matrix elements [*H̃*]_*l*,*l*′_ can be obtained as follows
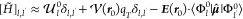
4where ***r***_0_ is the reference position of the center of mass of the QC,  is the electric potential exerted by the environment, *q*_*T*_ is the total charge of the QC, ***E*** is the perturbing electric field generated by the classical environment, and ***μ*^** is the electric dipole moment operator.

In the atom-based expansion, we can rewrite the perturbing electric potential within the Cartesian subspace including all of the QC nuclei and all of the spatial positions significantly accessible to the QC electrons, , by considering the atomic regions (i.e., the nonoverlapping spatial regions centered at each nucleus) while reasonably assuming that no relevant electronic density is present outside such regions. Therefore we obtain
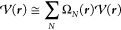
5where , *N* runs over all of the QC atoms, and each Ω_*N*_ is a step function being null outside the *N*th atomic region and equal to the unity inside. Then, by expanding the perturbing electric potential within each *N*th atomic region around the *N*th nucleus position ***R***_*N*_, and by inserting into eq 3, it provides
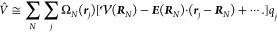
6For a QC with nonvanishing atomic charges, we can express the elements of the perturbed Hamiltonian matrix as
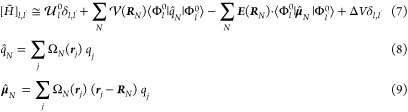
7where *q̂*_*N*_ and ***μ*^**_*N*_ are the *N*th atomic charge and dipole operator, respectively. In eq 7, we include explicitly the terms up to the dipolar contribution (with the difference, compared to the QC-based approximation, of considering the expansion within each small atomic region), while all of the terms beyond the atomic dipolar ones are included in the nuclear position function Δ*V*. It is worth noting that ⟨Φ_*l*_^0^|*q̂*_*N*_|Φ_*l*_^0^⟩ are the *l*th unperturbed electronic eigenstate atomic charges (unperturbed atomic charges) and generally the corresponding atomic dipoles (unperturbed atomic dipoles) can be neglected (i.e., ⟨Φ_*l*_^0^|***μ*^**_*N*_|Φ_*l*_^0^⟩ ≅ 0) since the electronic density of each *l*th unperturbed electronic eigenstate within each atomic region can be considered symmetric around the nucleus. Due to the difficulty involved in obtaining the atomic charge and dipole operators as expressed in the unperturbed electronic basis set involving different eigenstates (i.e., ⟨Φ_*l*_^0^|*q̂*_*N*_|Φ_*l*′_^0^⟩ and ⟨Φ_*l*_^0^|***μ*^**_*N*_|Φ_*l*′_^0^⟩ when *l* ≠ *l*′), instead of using eq 7 to construct the Hamiltonian matrix, we used the atom-based expansion only for the Hamiltonian matrix diagonal elements (for which the unperturbed atomic charges can be provided by commonly available quantum chemical calculations and the unperturbed atomic dipoles can be disregarded). In PyMM, all of the other Hamiltonian matrix elements are calculated using the QC-based expansion within the dipole approximation (eq 4). Therefore, we can write
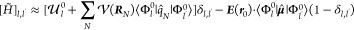
10

The default behavior of run_pmm is to perform an MD-PMM calculation within the QC-based expansion approximation. An atom-based expansion for the diagonal elements of the electronic Hamiltonian matrix (while keeping a QC-based expansion for the off-diagonal elements) can be performed by providing the atomic charges for each electronic state of the QC using the option -ch. The inputs needed for an MD-PMM calculation are as follows:

- the MD trajectory in a compatible file format (e.g., PDB, XTC, GRO).
- The classical atomic charges of the system used in the MD simulation (as a text file or using a compatible topology file, e.g., TPR type used in Gromacs).
- The reference QC geometry used for the QM calculation.
- The unperturbed electronic energies of the QC.
- The unperturbed electric dipole matrix of the QC.
- The QM atomic charges of each unperturbed electronic state of the QC (only necessary when the atom-based expansion approximation is requested).

At the end of the calculation, run_pmm outputs two files with the perturbed eigenvectors and eigenvalues for each frame of the trajectory, providing the necessary elements to calculate all of the perturbed electronic properties of the QC embedded in the environment.

### Perturbed Electronic Wavefunction Analysis: eig Module

**Figure 2:**
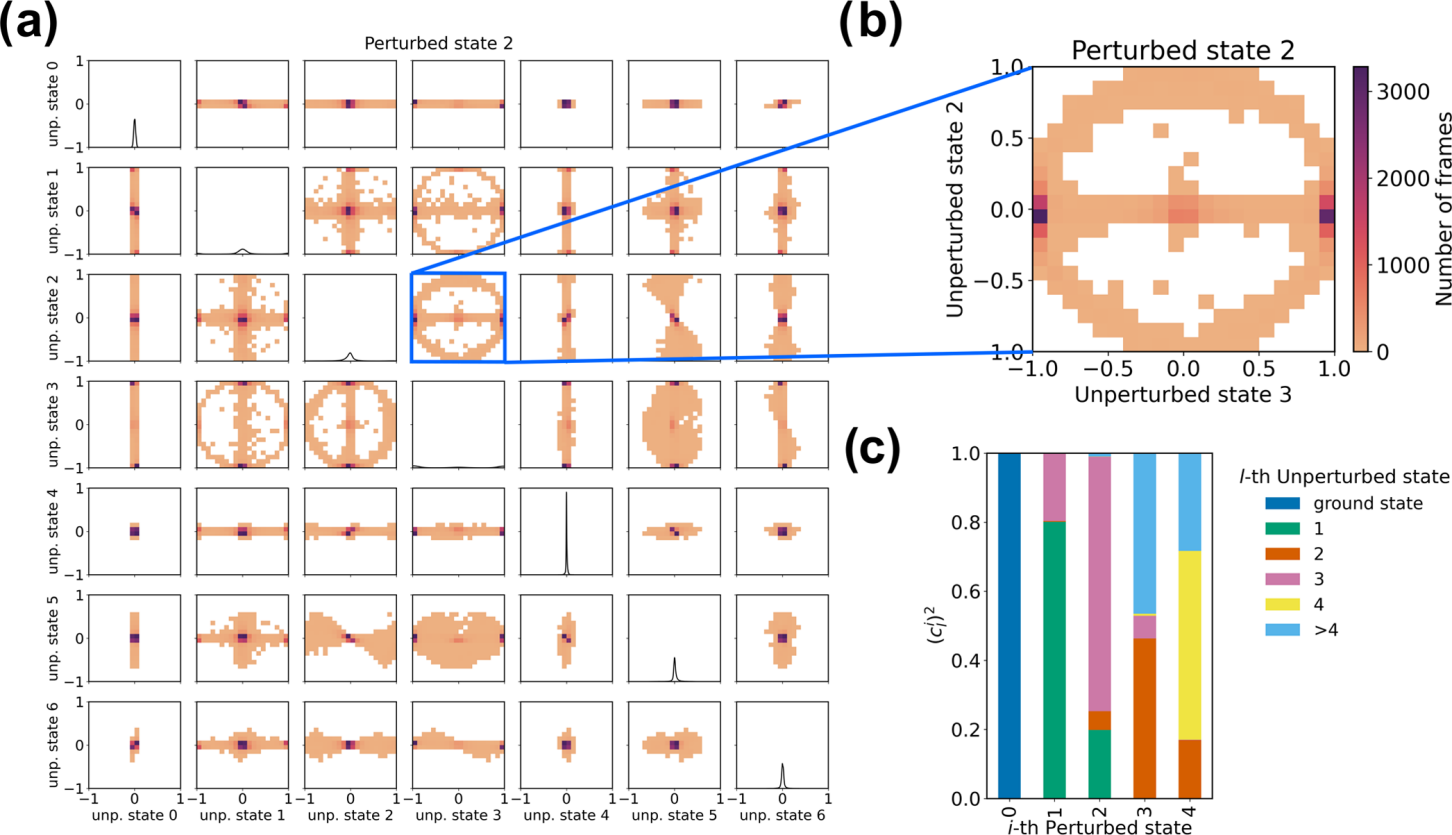
Example of analysis of the perturbed wavefunctions of indole in water solution (manuscript in preparation; unperturbed properties: EOM-CCSD/6-311++G(2d,2p); MD simulation: 949 SPC water molecules, CHARMM36m force field,^29^*T* = 300 K, 20 ns). (a) Total representation of the dynamical composition of the second perturbed excited electronic wavefunction expressed on the basis of the unperturbed states of indole. Each contribution arising from the unperturbed states is plotted against each other. (b) Details of the correlation between the second and third unperturbed states in the description of the second perturbed excited electronic state. It is possible to observe how the main contribution comes from the third unperturbed state, with minimal mixing with the second unperturbed state alone. Therefore, in solution, we can observe the inversion between those two states in large chunks of the simulation, while configurations in which the perturbed state is described as the mixing of the second and third unperturbed states are present albeit negligible. (c) Cumulative histograms representing the composition of the perturbed electronic states expressed on the basis of the unperturbed electronic states of indole, averaged over the entire simulation.

In the PMM framework, the *i*th perturbed electronic state wavefunction (Φ_*i*_) is expressed as a linear combination (with coefficients *c*_*l*_^*i*^) of the unperturbed electronic states (Φ_*l*_^0^)
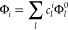
11pymm furnishes the dynamical description of the perturbed electronic wavefunctions along the MD simulation. A useful representation of the eigenvectors dynamical behavior is typically provided through their projection on the unperturbed basis set through the eig module. By plotting the value of the projections on each unperturbed state against each other for every frame of the simulation, it is possible to identify phenomena such as the change in the order of the electronic states due to the perturbation or recognize specific electronic configurations corresponding to different environment configurations as sampled along the MD simulation (see Figure 2a,b). PyMM can also provide the (averaged) contribution of the unperturbed states on the perturbed ones as a cumulative histogram (Figure 2c). Note that such a representation, although of immediate readability, does not account for instantaneous electronic configurations, which can be very important in describing the dynamical behavior of the perturbed electronic states.

### Prediction of Absorption Spectra: calc_abs Module

By means of MD-PMM, it is possible to calculate the UV–vis absorption spectra in liquid-state systems.^4,6,30^ In the case of a chromophore in solution that exhibits negligible vibronic transitions (i.e., vanishing vibrational overlap between ground to excited states) compared to the vertical one or when the vibronic vertical signal is broad enough to cover all of the other non-negligible vibronic transitions, the electronic vertical transition can provide a good approximation of the overall vibronic lineshape.^31,32^ The absorption spectrum of a chromophore in liquid-state system can be expressed through the extinction coefficient ε_0,*i*_(ν) for the 0 → *i* electronic transition, which can be calculated as follows
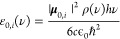
12where ϵ_0_ is the vacuum dielectric constant, *c* is the vacuum light speed, *h* is Planck’s constant, , |***μ***_0,*i*_|^2^ is the mean electronic transition dipole square length as obtained averaging within the ν, ν + dν frequency interval, and ρ = ρ(ν) is the probability density of the vertical transition frequency of the QC embedded in a the atomic-molecular environment. Using a proper and extensive sampling of the QC-environment statistical ensemble as provided by the *N* frames (in principle *N* → ∞) of the MD trajectory, it is possible to write
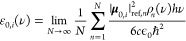
13where ρ_*n*_ = ρ_*n*_(ν) and |***μ***_0,*i*_|_ref,*n*_^2^ are the probability density of the transition frequency and the perturbed transition dipole square length, respectively, at the *n*th MD frame, for the reference configuration of the QC (note that both the transition frequency and the transition dipole are recomputed at each MD frame using the perturbed basis set). The ρ_*n*_ can be modeled by a Gaussian distribution centered in ν_ref,*n*_, i.e., the perturbed electronic vertical transition frequency of the *n*-th frame QC reference structure. Hence
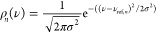
14with the variance, σ^2^, that can be reasonably estimated by a set of unperturbed electronic excitation each corresponding to a relevant QC configuration obtained from the MD sampling of the QC semiclassical vibrations. It is worth noting that the same variance is used for each MD frame, to simplify the computational procedure. Combining eq 14 in eq 12, the extinction coefficient can be calculated as follows:
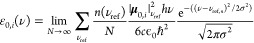
15

In the last equation, the sum runs over the perturbed vertical transition frequencies of the reference structure partitioned in a large number of tiny bins and *n*(ν_ref_) is the number of MD frames with electronic excitation frequency within the bin centered in ν_ref_. |***μ***_0,*i*_|_ν_ref__^2^ is the reference structure mean electronic transition dipole square length obtained by averaging over the bin centered in ν_ref_. The complete absorption spectrum can be obtained by summing all of the relevant spectral contributions identified by 0 → *i* electronic transitions in the spectral region of interest.

Following the procedure described above, the UV–vis spectrum as modeled by the MD-PMM appraoch can be calculated using the pymm calc_abs command. It requires the unperturbed electric dipole matrix, the eigenvalues, and the eigenvectors trajectories (as obtained by pymm run_pmm). The default value of σ (see eqs 14 and 15) is set to 0.034 eV, a value used in previous works for small–medium-sized organic molecules.^6−8^

### Prediction of Free Energies: free_en Module

The MD-PMM approach allows the evaluation of the free energy change between two electronic states corresponding to two chemical states, thus providing an efficient procedure to model, for example, the calculation of redox reaction free energy.^33^ Note that we disregard the quantum vibrational effects as these typically provide negligible reaction energy contributions compared to the electronic ones. Considering a general reactant *R* to product *P* reaction *R* → *P*, the corresponding Helmholtz free energy change Δ*A* providing the reaction free energy can be expressed as
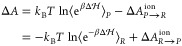
16where *k*_B_ is the Boltzmann constant, *T* is the temperature, β = (*k*_B_*T*)^−1^,  is the QC and environment energy change (i.e., the total energy change) upon the *R* → *P* transition, and the angular parentheses indicate the ensemble average (⟨···⟩_*P*_ for the product ensemble and ⟨···⟩_*R*_ for the reactant ensemble). Furthermore, Δ*A*_*P*→*R*_^ion^ is the relaxation free energy for the *R* species due to the *P* → *R* ionic environment transition and Δ*A*_*R*→*P*_^ion^ is the relaxation free energy for the *P* species due to the *R* → *P* ionic environment transition. Such contributions should be included when the reaction includes a change in the charge state of the QC. Assuming that the environment internal energy change associated with the reaction is negligible,  can be approximated by the QC electronic energy change as obtained by the *R* and *P* energy minima for the quantum vibrational coordinates, 
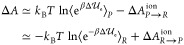
17

Assuming Δ*A*_*P*→*R*_^ion^ ≃ Δ*A*_*R*→*P*_^ion^, the following expression can be written
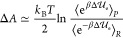
18

**Figure 3:**
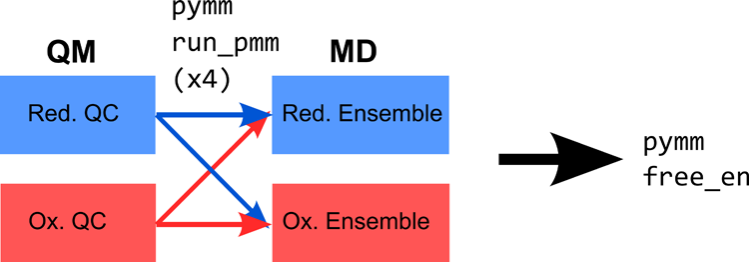
Diagram showing the steps required for the calculation of the reaction free energy, in the case of a redox process involving the reduced (Red.) and oxidized (Ox.) states corresponding to the *R* and *P* chemical states.

The last equation is used in the pymm free_en module for the calculation of the reaction free energy change. Equation 18 makes use of both the product and reactant ensembles, as provided by the MD simulations of the *R* and *P* species, respectively. For each MD frame of such simulations, two MD-PMM calculations need to be performed: one with the QC in the *R* form and the other with the QC in the *P* form (see Figure 3).

## Examples of Use

To show the capabilities of PyMM, we describe its application to two molecules in solution, doxorubicin (DX) and deoxyguanosine. The application of the PyMM to doxorubicin allowed us to model its perturbed electronic excited states and to accurately predict its absorption spectrum in water,^7^ whereas for the deoxyguanosine, we applied the PyMM to estimate the oxidation free energy (i.e., the oxidation potential) in solution.^10^ Note that in the test set included in the code, we also provide the data to evaluate the absorption spectrum of water by means of PyMM.^34,35^

### Doxorubicin

**Figure 4:**
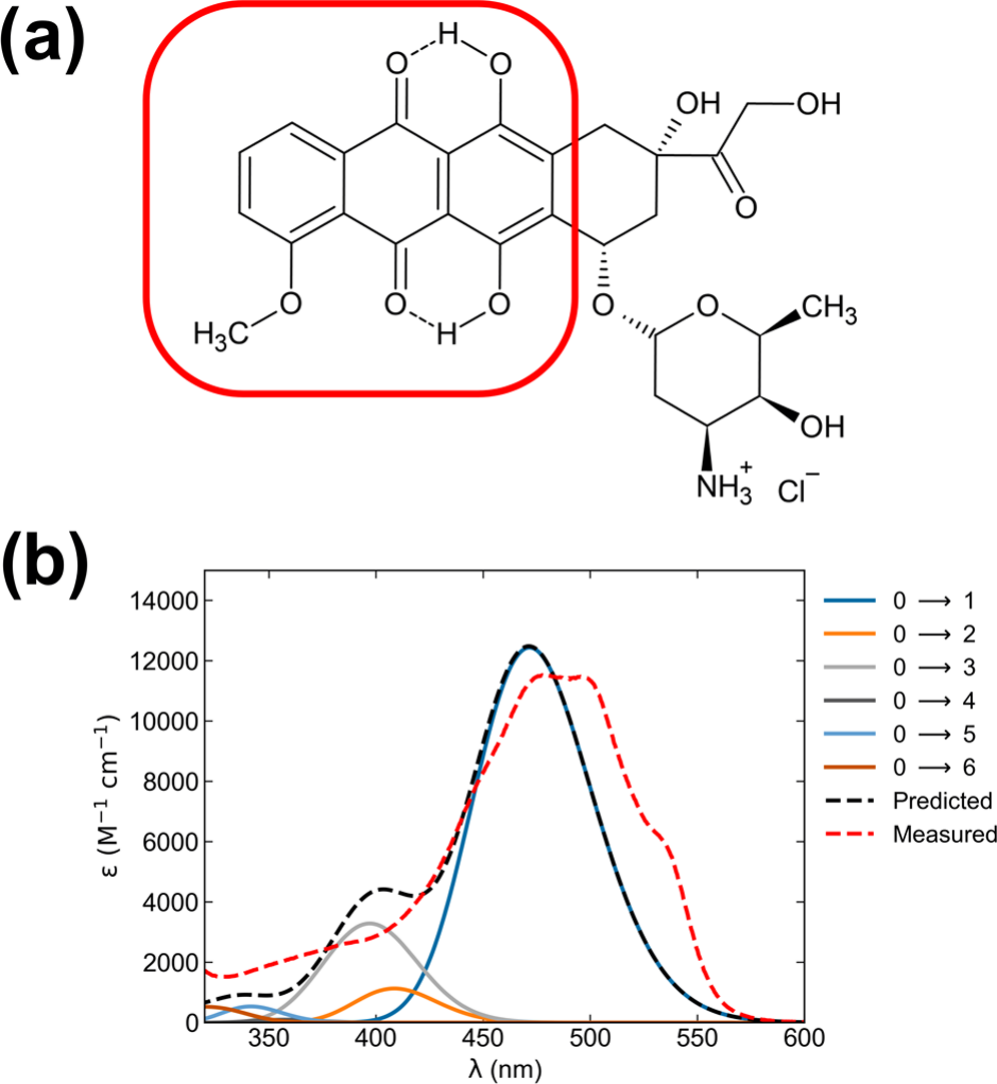
(a) Doxorubicin hydrochloride structure. The QC (1,4-dihydroxy-5-methoxy-9,10-anthraquinone) is indicated by the red rectangle. (b) Predicted absorption spectrum of DX in aqueous solution (black, dashed line) compared to the experimental spectrum (red, dashed line). The contributions arising from each calculated electronic transition are also reported in different colors.

**Figure 5:**
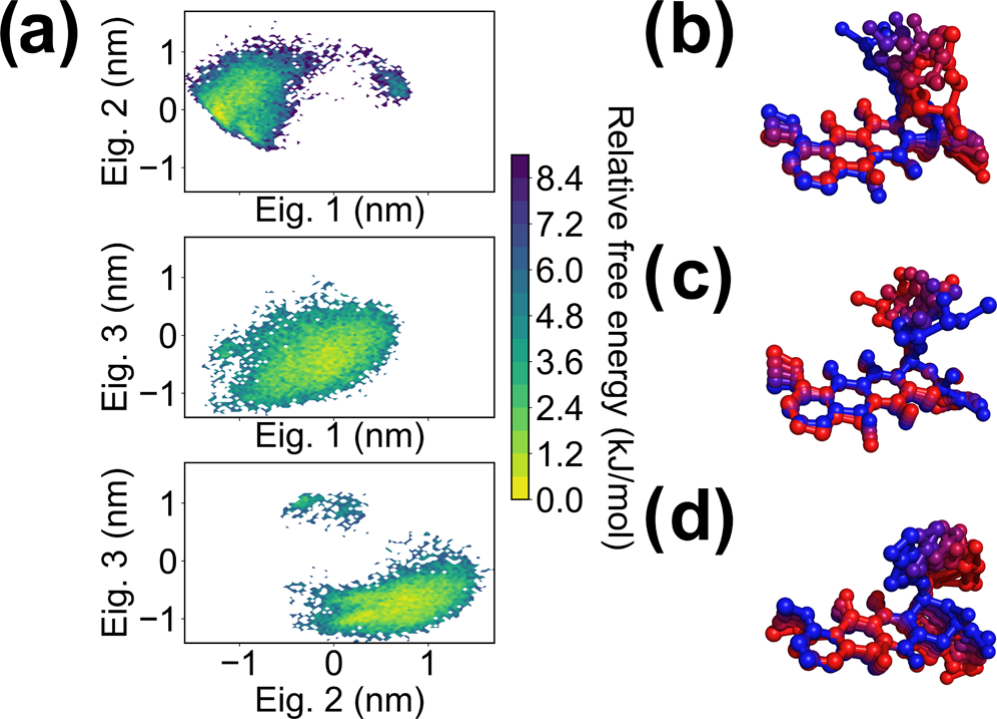
(a) Projections of the MD trajectories on the first, second, and third principal components (eigenvectors) of DX in water. The color map indicates the calculated relative free energy, with the minimum sampled value set as the reference. (b–d) Images representing the concerted motions described by the eigenvectors 1 (b), 2 (c), and 3 (d) by overlapping frames extracted from the MD trajectory ordered according to the values of their projections on the eigenvectors.

The properties of the first 11 unperturbed electronic states of the QC of DX (in this case, 1,4-dihydroxy-5-methoxy-9,10-anthraquinone; Figure 4a) were obtained using the TD-DFT level of theory (B3LYP/6-311++G(2d,2p)) as provided by the Q-Chem^23^ software. Such a QC allowed to account for the conformational freedom of DX (which are mainly due to the 2-hydroxyacetyl group and the oxane moiety, as determined by the Essential Dynamics;^36^ see Figure 5) on the classical level, thus limiting the number of QM calculations necessary to describe the internal degrees of freedom of the molecule. The MD simulation of 150 ns at *T* = 300 K was carried out by means of Gromacs software package^16^ using a DX molecule placed in a cubic box of side 6.301 nm with 8309 SPC water molecules and a Cl^–^ anion. A timestep of 2 fs was used. The force field parameters for DX were obtained using the Automated Topology Builder (ATB).^37^ The MD-PMM calculation was performed at the QC-based expansion level of approximation using pymm run_pmm, and the UV–vis spectra were obtained using the pymm calc_abs module (Figure 4b).

**Figure 6:**
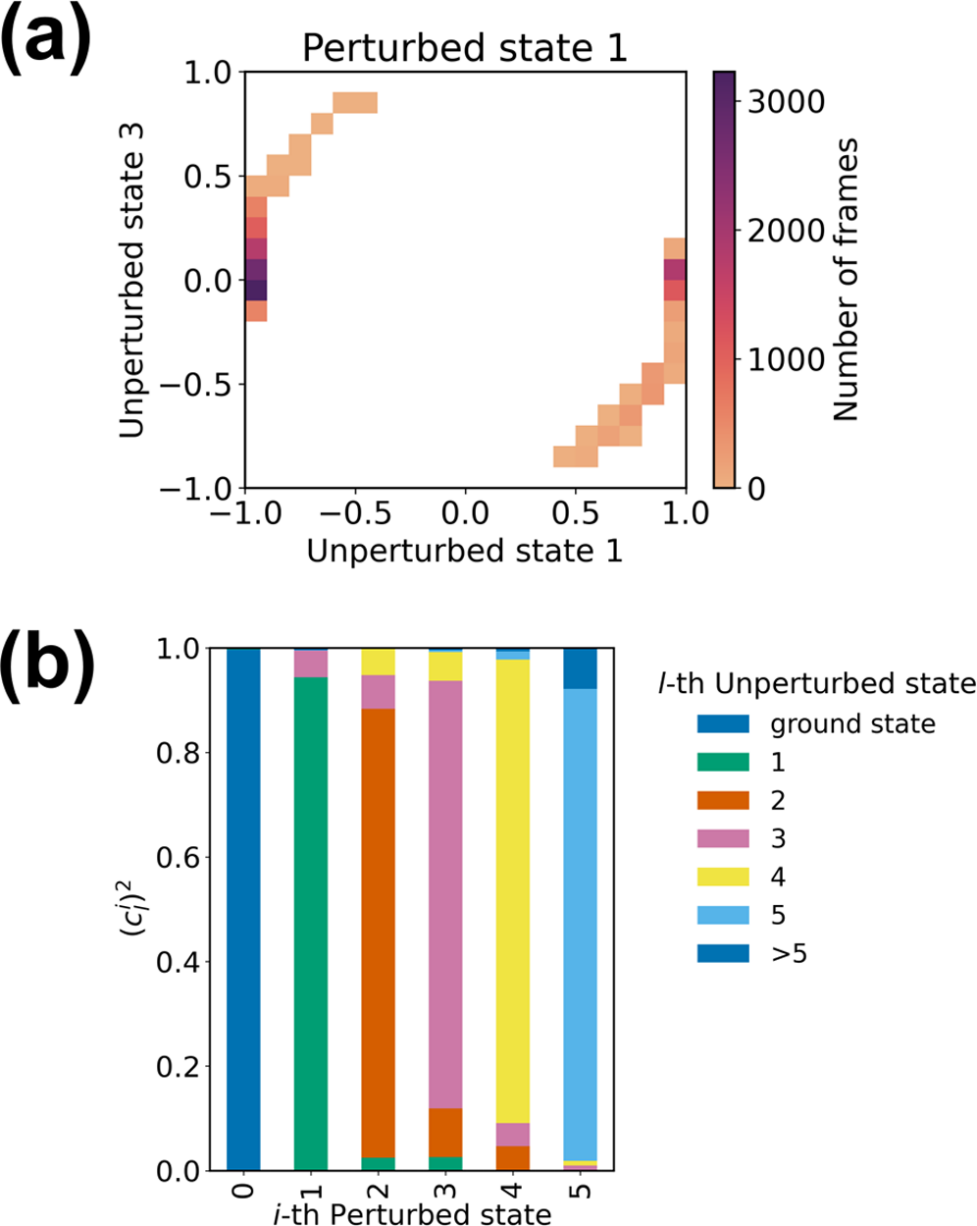
(a) Main contributions (i.e., the coefficients of the first and third unperturbed states in the expansion of the perturbed electronic wavefunction) to the first perturbed electronic state of DX plotted against each other. (b) Cumulative histograms representing the composition of the first six perturbed electronic states expressed on the basis of the unperturbed electronic states of DX, averaged over the entire simulation.

From the analysis of projections on the unperturbed electronic states for the first perturbed eigenvector, it was possible to describe the effect of the perturbation provided by the solvent and how it causes a limited mixing between the first and third unperturbed electronic states (Figure 6a). In fact, the first perturbed electronic states remain mostly unchanged with respect to the unperturbed ones along the simulation, i.e., they can be approximated rather accurately by the corresponding unperturbed electronic states (see Figure 6b).

### Deoxyguanosine

The properties of the ground and the first seven excited states of the QCs, i.e., the guanine base, in the neutral and radical cation states, were obtained using Gaussian software package,^21^ at TD-DFT (B3LYP/6-311++G(2d,2p)) level of theory. MD simulations for the neutral deoxyguanosine (neutral ensemble) and for the ionized deoxyguanosine (oxidized ensemble) were carried out in a cubic box of 3.1 nm sides filled with 1074 SPC water molecules at 300 K for 100 ns using Gromacs software package^16^ and AMBER99sb-ildn force field.^38^ For the simulation of the ionized deoxyguanosine, the atomic partial charges were estimated by the same procedure used for the estimation of the parameters in the AMBER force field.^38^ Additional simulations were performed in acetonitrile^39^ for the evaluation of the solvent effect on the oxidation properties of the deoxyguanosine in solution. The MD-PMM calculation was performed both at the QC-based and atom-based expansion level approximation using pymm run_pmm, and the reduction free energy changes were obtained using pymm free_en module. The equation:  was used to calculate the reduction potential of the deoxyguanosine cation in aqueous and acetonitrile solution with respect to the standard hydrogen electrode (Table 1). The value of 4.281*V* for *E*_SHE_^0^ was used.

**Table 1:** Calculated Reduction Free Energy Changes and Reduction Potentials of Ionized Deoxyguanosine in Aqueous and Acetonitrile Solution

	Δ*A*^a^	*E*^0^^b^,^c^
	Water sol.	
QC-based expansion	–489.1 kJ/mol	0.79 V
atom-based expansion	–497.7 kJ/mol	0.87 V
	Acetonitrile sol.	
QC-based expansion	–510.8 kJ/mol	1.01 V
atom-based expansion	–495.4 kJ/mol	0.86 V

a The estimated standard error on the reduction free energy is ±5.0 kJ/mol.

b The estimated standard error on the calculated reduction potential in acetonitrile is ±0.05 V.

c Values are reported against SHE.

**Figure 7:**
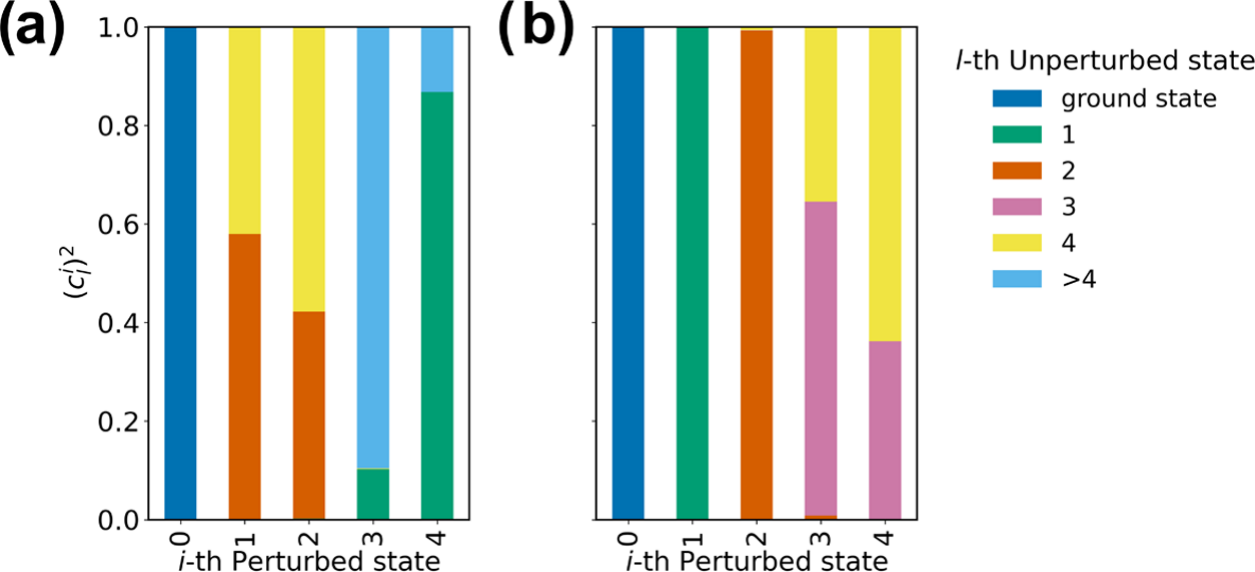
Cumulative histogram representing the square projections of each perturbed eigenvectors of the guanine QC in the neutral (a) and radical cationic (b) forms on their corresponding sets of unperturbed electronic states averaged over the sampled frames. Only the first five states were considered explicitly.

The analysis of the perturbed eigenvectors, provided by MD-PMM, shows that the considered reaction involves electronic states of the reduced and oxidized species, which can be approximated to their corresponding unperturbed ground states (i.e., no contribution from the unperturbed excited states to the perturbed ground state were observed, see Figure 7).

**Figure 8:**
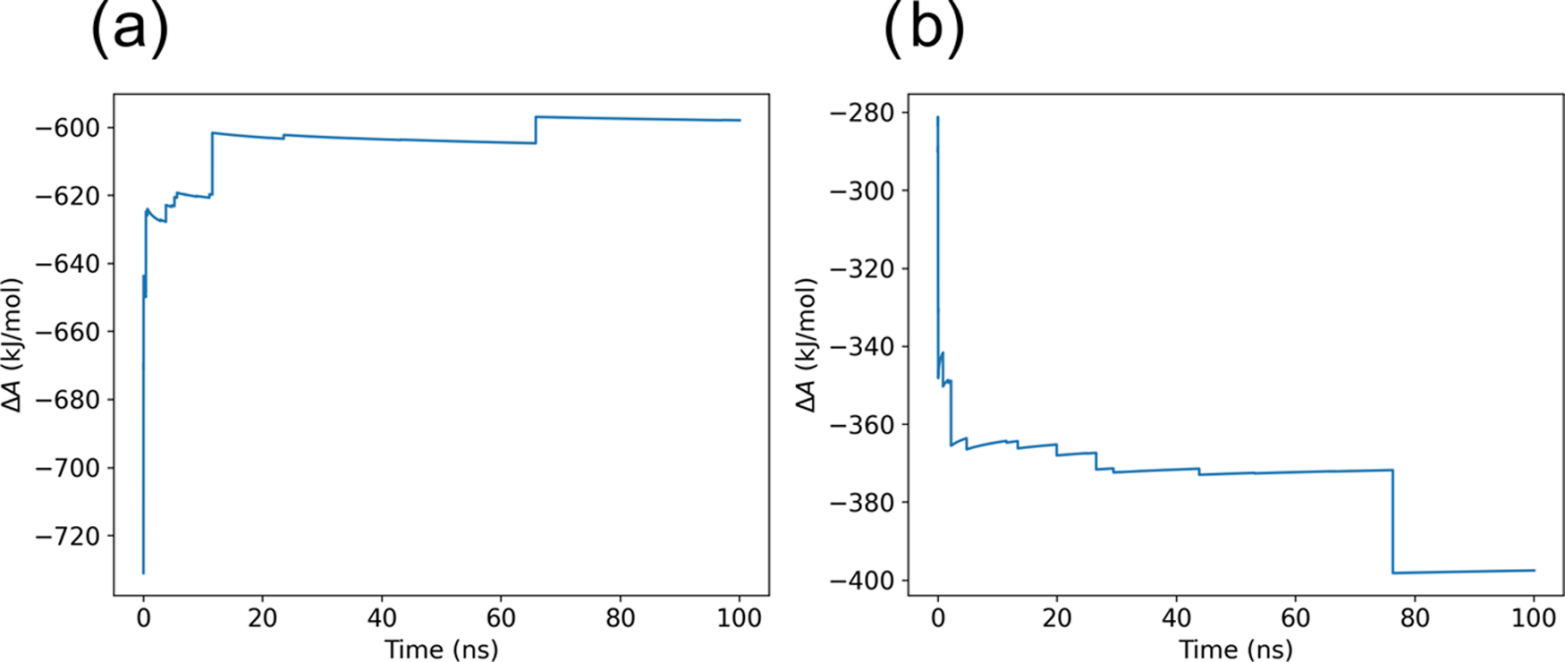
Reduction free energy of ionized deoxyguanosine in water as a function of the number of frames used for its evaluation (a) for the neutral ensemble and (b) for the oxidized ensemble.

The reduction free energy (Δ*A*) convergence was checked by calculating Δ*A* as a function of the number of MD trajectory frames used in both the neutral and oxidized ensembles (see Figure 8).

Such calculations were repeated in more complex nucleic acids to accurately describe the conformational and sequence effects.^40^

## Availability

PyMM is freely and publicly available in our repository on GitHub (https://github.com/ChenGiuseppe/PyMM) under the GNU General Public License version 2.

Table 2 shows the systems and the main libraries version on which PyMM has been tested.

**Table 2:** Systems on Which PyMM Has Been Tested

operative system	Python	MDAnalysis
Linux	3.9, 3.10	2.3.0
Windows 10/11	3.9, 3.10	2.3.0
MacOS	3.9, 3.10	2.3.0

## Future Developments

Support to other types of calculations in the MD-PMM framework such as the prediction of the emission,^8^ IR,^4,9^ CD,^6^ and vibronic^8^ spectra, and the modeling of excitonic effects between interacting chromophores,^41^ are currently in development.
